# Association between MTHFR C677T polymorphism and abdominal aortic aneurysm risk: A comprehensive meta-analysis with 10,123 participants involved

**DOI:** 10.1097/MD.0000000000004793

**Published:** 2016-09-09

**Authors:** Jie Liu, Xin Jia, Haifeng Li, Senhao Jia, Minhong Zhang, Yongle Xu, Xin Du, Nianrong Zhang, Weihang Lu, Wei Guo

**Affiliations:** a Department of Vascular and Endovascular Surgery, Chinese PLA General Hospital, Beijing, China; b Department of Vascular and Endovascular Surgery, Shanxian Dongda Hospital, Heze, Shandong; c Department of Nephrology, Chinese-Japan Friendship Hospital, Beijing, China.

**Keywords:** abdominal aortic aneurysm, homocysteine, metaanalysis, methylenetetrahydrofolate reductase

## Abstract

**Background:**

Abdominal aortic aneurysm (AAA) is a life-threatening condition. A number of studies reported the association between methylenetetrahydrofolate reductase (MTHFR) C677T polymorphism and AAA risk, but substantial controversial findings were observed and the strength of the association remains unclear.

**Objective:**

The aim of this study was to investigate the aforementioned association in the overall population and different subgroups.

**Methods:**

PUBMED and EMBASE databases were searched until March 2016 to identify eligible studies, restricted to humans and articles published in English. Summary odds ratios (ORs) and 95% confidence intervals (CIs) were used to evaluate the susceptibility to AAA. Subgroup meta-analyses were conducted on features of the population, such as ethnicity, sex of the participants, and study design (source of control).

**Results:**

Twelve case–control studies on MTHFR C677T polymorphism and AAA risk, including 3555 cases and 6568 case-free controls were identified. The results revealed no significant association between the MTHFR C677T polymorphism and AAA risk in the overall population and within Caucasian or Asian subpopulations in all 5 genetic models. Further subgroup meta-analysis indicated that significantly increased risks were observed among cases with a mean age <70 years (OR = 1.73, 95% CI = 1.10–2.12, *P* = 0.02), cases with prevalence of smoking <60% (OR = 1.39, 95% CI = 1.02–1.90, *P* = 0.04), and cases with aneurysm diameter ≥55 mm (OR = 1.55, 95% CI = 1.07–2.24, *P* = 0.02) in the dominant genetic model. No publication bias was detected in the present study.

**Conclusion:**

In conclusion, our comprehensive meta-analysis suggests that the MTHFR C677T polymorphism may play an important role in AAA susceptibility, especially in younger, non-smoking, larger AAA-diameter subgroups of patients

## 1 Introduction

Abdominal aortic aneurysm (AAA) is a life-threatening condition detected in up to 9% of men ages >65 years in Western populations.^[1–4]^ Several risk factors are recognized, such as age >65 years, maleness, smoking, and a positive family history of AAAs. Protective factors include type-2 diabetes mellitus, African-American ethnicity, and femaleness.^[2,5,6]^ Although many risk factors have been identified, the etiology of AAA is still largely unclear.^[7,8]^

Homocysteine (Hcy) is a sulfur-containing nonessential amino acid that functions as a key intermediate in the methionine metabolism. Methylenetetrahydrofolate reductase (MTHFR) is a key enzyme involved in Hcy metabolism. A common functional polymorphism (C677T) in the gene-encoding MTHFR results in a 70% reduction in enzymatic activity, *in vitro*, and has been associated with differences in Hcy concentration.^[9,10]^ Previous studies on the MTHFR C677T polymorphism suggested a causal link between hyperhomocysteinemia (HHcy), characterized by elevated Hcy levels in the blood, coronary heart disease, stroke, and peripheral vascular disease.^[11,12]^

However, studies investigating the association between HHcy and AAA yielded conflicting results.^[13–20]^ Whether Hcy levels play a causal role in the pathogenic process of AAA remains unclear. To overcome the problem of reverse causality, studies have focused on the association between the MTHFR C677T polymorphism and AAA risk. However, the association is still inconsistent. Although 5 case–control studies ^[13–15,21,22]^ showed that carriers of the MTHFR 677T allele had an increased risk of AAA, 7 others^[17,18,20,23–26]^ showed no significant association between these 2 factors. Furthermore, 4 studies^[25,27–29]^ investigating this association using forest plots also provided conflicting results. The present study was designed to investigate the association between the MTHFR C677T polymorphism and AAA risk by performing a comprehensive meta-analysis, including a subgroup analysis.

## 2 Materials and methods

### 2.1 Publication search

Two independent investigators performed a comprehensive search for all studies on the association between the MTHFR C677T polymorphism and AAA risk in 2 electronic databases (PubMed and Embase) through March 31, 2016. We used the search terms “mthfr,” “methylenetetrahydrofolate reductase,” “5,10 methylenetetrahydrofolate reductase,” “677C,” “C677T,” and “rs1801133” in combination with “aneurysm.” We searched for any additional studies in the references of all identified publications, including previous relevant meta-analyses. Unpublished data and supplemental information were also requested from authors. The search was restricted to studies in humans and articles published in English. This study protocol was approved by the ethics committee of Chinese PLA General Hospital.

### 2.2 Selection and exclusion criteria

Two investigators independently screened all titles and abstracts of the identified studies. Only studies published as full-length articles or letters in peer-reviewed journals were included. Studies included were in accordance with the following criteria: original case–control studies with matching control subjects; clinical trials studying the association between the MTHFR C677T polymorphism and AAA risk; all cases were diagnosed by ultrasound or computed tomography angiography; contained at least 2 comparison groups (AAA group and control group); all genotype distributions were reported in both case and control groups. Accordingly, the following exclusion criteria were also used: no controls; insufficient data reported (eg, abstracts, editorial, comments, reviews, and meta-analyses); and studies with duplicated data.

### 2.3 Data extraction and synthesis

For each study, data were extracted using a standard form by 2 independent authors. For each included study, the following information was collected: name of the first author; year of publication; country of origin, ethnicity, and sex of the participants; study design (source of control); numbers of cases and controls; mean age in case groups; prevalence of smoking in case groups; mean diameter of aneurysms; and numbers of CC, CT, and TT genotypes in cases and controls. In the event of a disagreement between authors, discrepancies of included studies were resolved by a third author.

### 2.4 Statistical analysis

We carried out this meta-analysis referring to PRISMA guidelines. The associations between the MTHFR C677T polymorphism and AAA were calculated as the pooled odds ratio (OR) with its corresponding 95% confidence interval (CI) in the allele contrast (T vs C), homozygote (TT vs CC), heterozygote (CT vs CC), recessive (TT vs CT + CC), and dominant (TT + CT vs CC) genetic models. The significance of the pooled OR was determined by the *Z* test, and a *P* value <0.05 was considered significant. We performed subgroup-analysis according to ethnicity (Caucasian or Asian), sex of the participants (females or not), source of control (hospital or population), mean age in case groups (≥70 years or <70 years), prevalence of smoking in case groups (≥60% or <60%), and mean diameter of aneurysm (≥55 mm or <55 mm).

Heterogeneity was assessed by the chi-square–based Cochran *Q* test and the *I*^2^ statistic. Heterogeneity was regarded as statistically significant if *P*_het_ < 0.10 or *I*^2^ > 50%. The random-effects model was used if the test of heterogeneity was significant. Otherwise, the fixed-effects model would be applied in the analysis. To assess the stability of the results, sensitivity analyses were performed to determine whether the exclusion of any studies could affect the results. The potential publication bias was primarily appraised by Begg funnel plot and further evaluated using the Egger test ^[30]^ with STATA 12.1 software (Stata Corporation, College Station, TX).

EpiData 3.1 (EpiData Association, Odense, Denmark) was used to gather initial data in a standard form, and Endnote X7 (Thomson Reuters [Scientific] Inc., Carlsbad, CA) was used for literature management. All statistical analyses were carried out with the review manager (RevMan 5.3 The Cochrane Collaboration, Oxford) and the STATA software version 12.1 (Stata Corporation, College Station, TX). All *P* values in the meta-analysis were 2-tailed, and *P* values <0.05 were considered significant.

## 3 Results

### 3.1 Characteristics of studies

**Figure 1:**
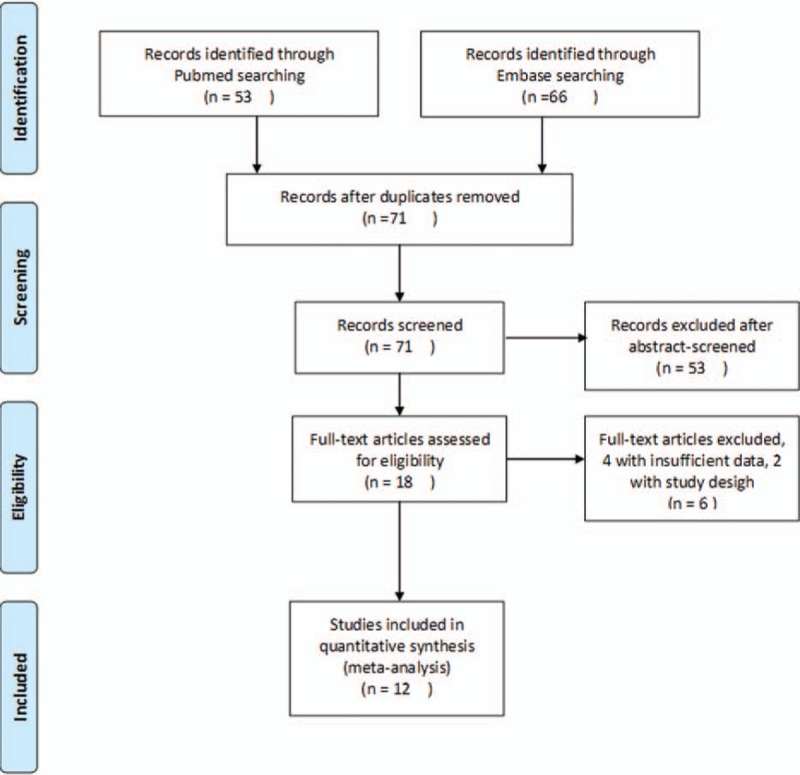
Flowchart of study selection referring to PRISMA guideline.

Using the Pubmed and Embase databases, 119 studies were identified. After excluding 48 duplicates, 71 studies were identified for further evaluation. After title and abstract screening, 53 irrelevant studies were excluded. After assessing the remaining 18 full-text articles, 4 studies were excluded for insufficient data, and 2 for not being case–control studies (Fig. 1).

Ultimately, a total of 12 case–control studies,^[13–15,17,18,20–26]^ including 3671 cases and 6569 controls, were identified. The characteristics of the 12 included studies are presented in Tables 1 and 2. Ten studies^[13,14,17,18,21–26]^ were based on Caucasian populations, and 2 studies^[15,20]^ were based on Asian populations. Five studies^[17,21–23,26]^ were population-based case–control studies, another 5^[13,15,24,25]^ were hospital-based, 1^[14]^ did not clarify the source of controls, and 1^[20]^ was a combination (hospital and population based). Ten studies^[13,15,18,20–26]^ included both males and females and the remaining 2^[14,17]^ included only males. In 6 studies,^[14,15,20–22,25]^ the mean age of the case group was <70 years. In 5 studies,^[13–15,21,26]^ the prevalence of smoking in the case group was <60%. In 6 studies,^[13,15,21–23,25]^ the mean diameter of aneurysm was >55 mm.

**Table 1:**
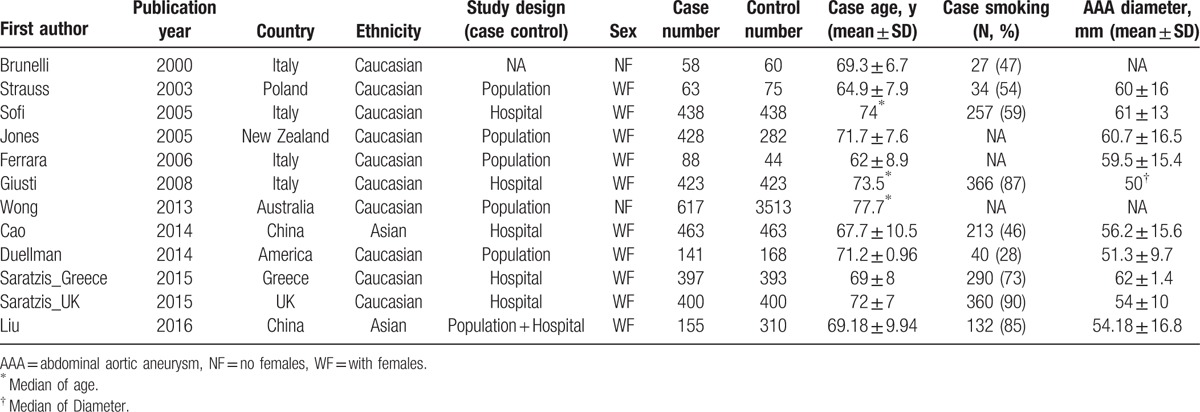
Characteristics of the studies included in the meta-analysis.

**Table 2:**
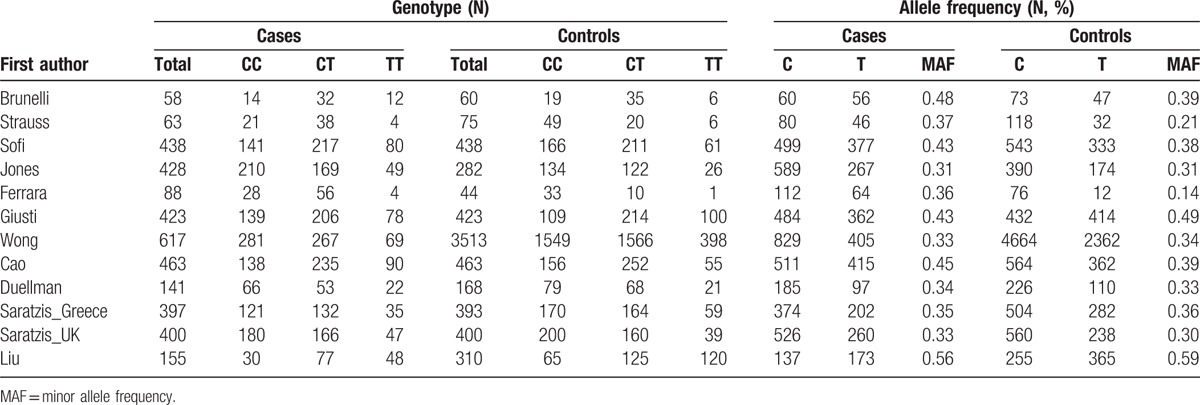
MTHFR C677T polymorphism genotype distribution and allele frequency in cases and controls.

### 3.2 Meta-analysis results

The frequency of the T allele varied widely across the 12 studies, ranging from 0.21 to 0.59 (Table 2). The average frequency of the T allele in overall populations was 0.37. There was a significant difference in frequency between Caucasians and Asians (0.34 vs 0.50, *P* < 0.01).

**Figure 2:**
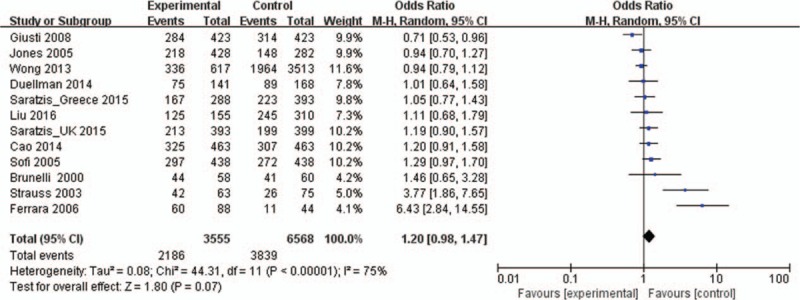
Forest plots of MTHFR C677T gene polymorphism and abdominal aortic aneurysm risk in the overall population (TT + CT vs CC). The squares and horizontal lines correspond to the study-specific OR and 95% CI. The area of the squares reflects the weight (inverse of the variance). The diamond represents the summary OR and 95% CI. CI = confidence interval, MTHFR = methylenetetrahydrofolate reductase, OR = odds ratio.

**Figure 3:**
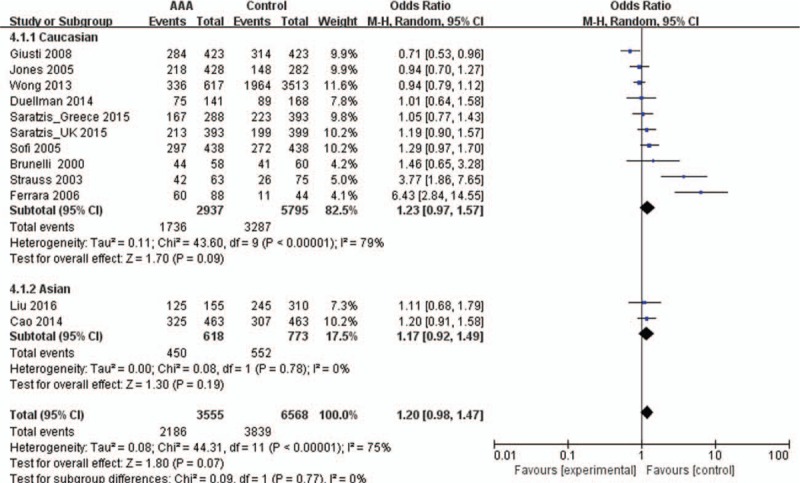
Forest plots of MTHFR C677T gene polymorphism and abdominal aortic aneurysm risk in the Asian and Caucasian population (TT + CT vs CC). The squares and horizontal lines correspond to the study specific OR and 95% CI. The squares and horizontal lines correspond to the study-specific OR and 95% CI. The area of the squares reflects the weight (inverse of the variance). The diamond represents the summary OR and 95% CI. CI = confidence interval, MTHFR = methylenetetrahydrofolate reductase, OR = odds ratio.

The main results of the meta-analysis are listed in Table 3. Overall, there was no association between the variant genotypes and AAA risk in different genetic models when all 12 studies were pooled into the meta-analysis, as shown in Table 3 and Fig. 2 (T vs C: OR = 1.12, 95% CI = 0.98–1.29, *P* = 0.10; TT vs CC: OR = 1.16, 95% CI = 0.91–1.47, *P* = 0.11; CT vs CC OR = 1.03, 95% CI = 0.99–1.08, *P* = 0.19; TT + CT vs CC: OR = 1.20, 95% CI = 0.98–1.47, *P* = 0.07; TT vs CT + CC: OR = 1.10, 95% CI = 0.89–1.35, *P* = 0.39). Ten studies^[13,14,17,18,21–26]^, including 2937 cases and 5795 controls, were used to investigate the association in Caucasian populations. The results showed no association between the MTHFR C677T polymorphism and AAA risk in Caucasians using all 5 genetic models (Table 3 and Fig. 3). Two studies^[15,20]^ were designed to investigate the association in Asians, which also showed no association in all 5 genetic models (Table 3 and Fig. 3).

**Table 3:**
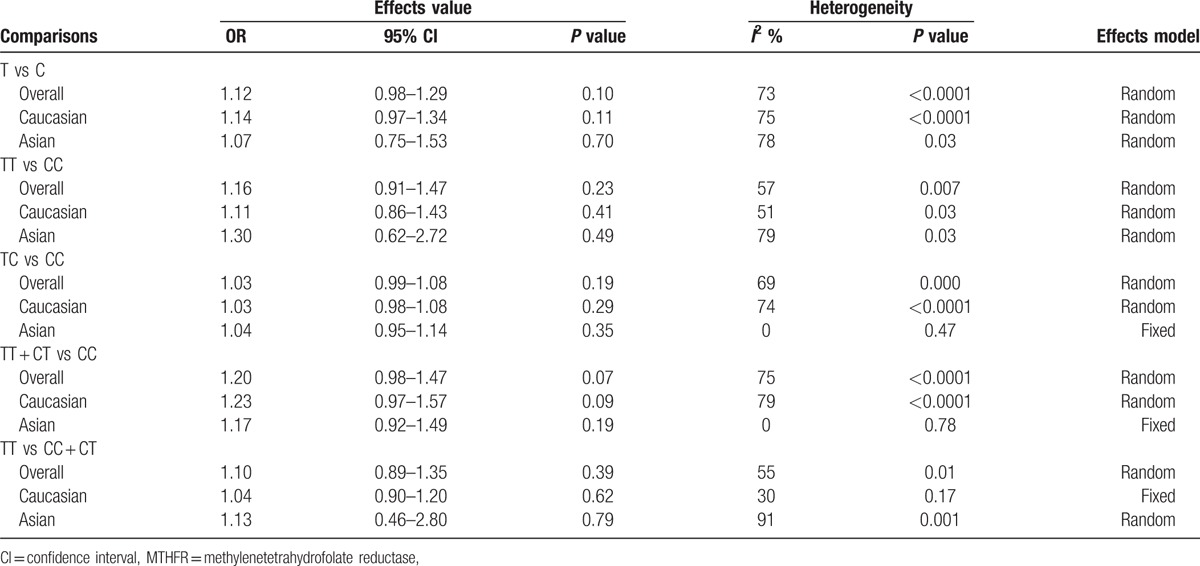
Meta-analysis of the association between MTHFR C677T polymorphism and abdominal aortic aneurysm risk based on different models.

The subgroup meta-analyses are shown in Table 4. Six studies^[14,15,20–22,25]^ with mean age <70 years old in the case group, including 1115 cases and 1345 controls, showed a significant association between the MTHFR C677T polymorphism and AAA risk in the dominant model (TT + CT vs CC: OR = 1.73, 95% CI = 1.10–2.12, *P* = 0.02). Five studies^[13–15,21,26]^ with prevalence of smoking in the case group <60, including 1163 cases and 1204 controls, showed a significant association in the dominant model (TT + CT vs CC: OR = 1.39, 95% CI = 1.02–1.90, *P* = 0.04). Six studies^[13,15,21–23,25]^ with AAA diameter ≥55 mm, including 1768 cases and 1695 controls, showed a significant association based on the dominant models (TT + CT vs CC: OR = 1.55, 95% CI = 1.07–2.24, *P* = 0.02).

**Table 4:**
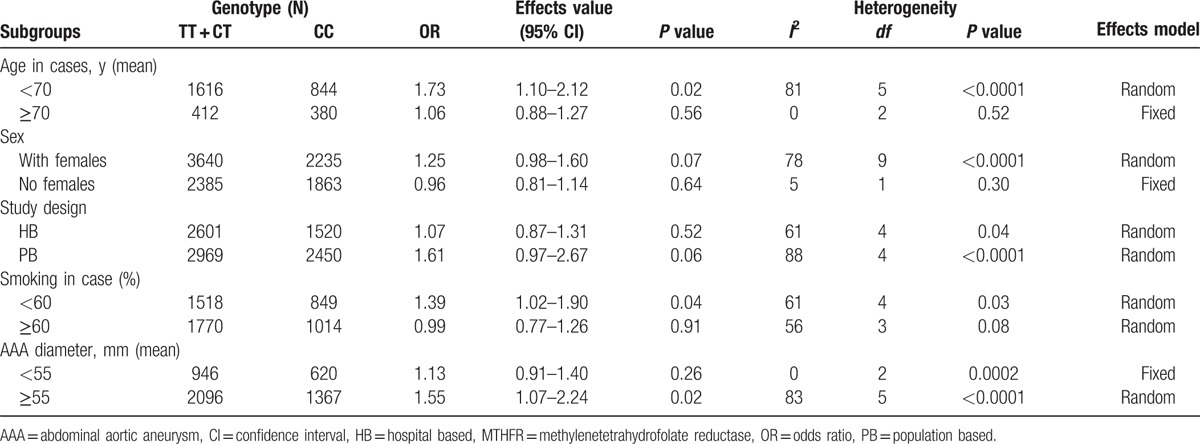
Subgroup meta-analysis of the association between MTHFR C677T polymorphism and abdominal aortic aneurysm risk based on the dominant models.

### 3.3 Publication bias

**Figure 4:**
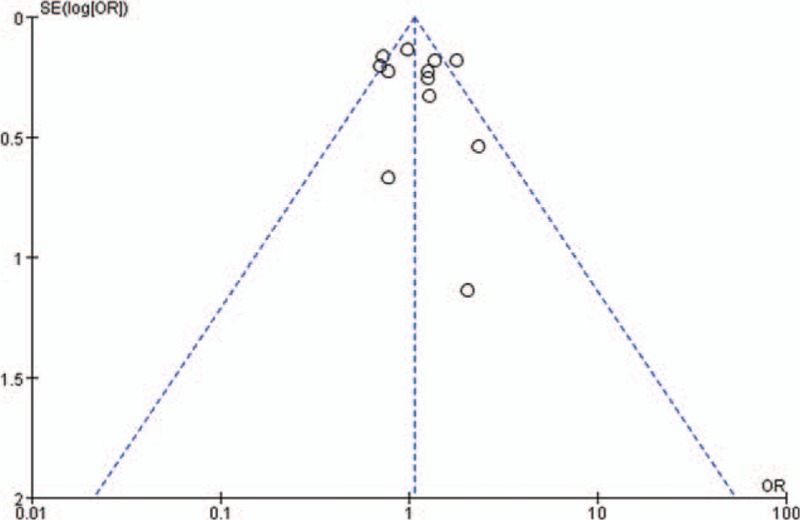
Funnel plot assessing evidence of publication bias from 12 studies (TT vs CT + CC).

A Begg funnel plot and an Egger test were performed to assess the publication bias. As shown in Fig. 4, the funnel plots did not reveal any obvious asymmetry in overall population, and the results of Egger test revealed no publication bias (*P* > 0.05).

## 4 Discussion

HHcy is an independent risk factor for the development of atherosclerosis, cardiovascular disease, and stroke.^[11,23]^ However, the results of previous studies^[13–18,20,24,31]^ that investigated the relationship between HHcy and AAA are inconsistent. Whether HHcy plays a direct causal role in the pathogenesis of AAA remains elusive. To overcome the problem of reverse causality, several studies^[13–15,17,18,20–26]^ sought an association between MTHFR genotype and AAA risk. However, the association between the MTHFR C677T polymorphism and AAA is still controversial.

In the present meta-analysis with 3555 cases and 6568 controls from 12 independent studies, no significant association was detected between the MTHFR C677T polymorphism and AAA risk in the overall population in all 5 genetic models. Additionally, no significant association was revealed within Caucasian or Asian subpopulations. However, the subgroup meta-analysis showed a significant association between the MTHFR C677T polymorphism and AAA risk in the subgroups of cases with a mean age <70 years, prevalence of smoking <60%, and AAA diameter ≥55 mm in the dominant genetic model. The results suggest that age, smoking, and AAA diameter may play a role in the relationship between the MTHFR C677T polymorphism and AAA risk.

Although 4 studies^[25,27–29]^ using forest plots investigated the association between the MTHFR C677T polymorphism and AAA risk, the results of these studies were controversial and lacked a subgroup analysis. Three meta-analyses^[25,27,28]^ showed that carriers of the MTHFR 677T allele presented with an increased risk of AAA. The study by Thompson et al,^[27]^ including 1075 cases and 1067 controls from 5 independent studies, revealed a significant effect of the T allele variant in increasing the risk of AAA (risk ratio = 1.14, 95% CI = 1.08–1.21). McColgan et al,^[28]^ including 817 cases and 629 controls from 6 studies (including 3 studies with the same author indicating a potential duplication of data), showed a similar significant result (TT + CT vs CC, OR = 1.34, 95% CI = 1.08–1.65). Saratzis et al,^[25]^ including 2498 cases and 6212 controls from 9 studies, also showed a significant OR between the MTHFR C677T polymorphism and AAA (OR = 1.07; 95% CI = 1.02–1.12). The meta-analysis ^[29]^ by Bradley et al, including 2498 cases and 6212 controls from 9 studies, showed no significant association between the MTHFR C677T polymorphism and AAA (Heterozygous [C/T], OR = 1.06, 95% CI = 0.87–1.30; Homozygous (T/T), OR = 1.06; 95% CI = 0.81–1.40). Our study, including 3555 cases and 6568 controls from 12 studies also showed no significant association between the variant genotypes and AAA risk in the 5 different genetic models. Furthermore, our meta-analysis of 10 studies^[13,14,17,18,21–26]^ in Caucasians, including 2937 cases and 5795 controls, showed no association between AAA risk and the variant genotypes in the different genetic models. Two studies in Asians^[15,20]^ also showed no association in all genetic models. Our results indicated that there is no significant association between the MTHFR C677T polymorphism and AAA in the overall population (both Caucasians and Asians).

However, the characteristics of included populations vary among different studies; the possibility of high heterogeneity and publication bias should be considered in a meta-analysis. Furthermore, a comprehensive meta-analysis requires the analysis of subgroups.

In our subgroup meta-analysis, the subgroup of 6 studies^[14,15,20–22,25]^ with a mean age <70 years in case groups, showed a significant association between the MTHFR C677T polymorphism and AAA risk in the dominant model (TT + CT vs CC, OR = 1.73, 95% CI = 1.10–2.12, *P* = 0.02). Ferrara et al^[22]^ divided the patients of the AAA group into 2 groups according to age and determined that the frequency of the 677T allele in younger patients with AAA was significantly higher than that in older patients with AAA. Our subgroup meta-analysis of 5 studies^[13–15,21,26]^ of cases with prevalence of smoking <60% also showed a significant association between the MTHFR C677T polymorphism and AAA risk (TT + CT vs CC, OR = 1.39, 95% CI = 1.02–1.90, *P* = 0.04). Strass et al^[32]^ divided the patients of the AAA group into 2 groups—those who smoke and those who do not—and showed that the frequency of the 677T allele in patients with AAA who smoke was higher than that in patients with AAA who do not, but this was not significant. In our subgroup meta-analysis of 6 studies^[13,15,21–23,25]^ of AAA with a diameter ≥55 mm, a significant association between the MTHFR C677T polymorphism and AAA risk was observed (TT + CT vs CC: OR = 1.55, 95% CI = 1.07–2.24, *P* = 0.02). Jones et al^[23]^ showed AAA diameter was significantly greater in T homozygotes compared to that in C allele carriers and the OR for T homozygotes and aneurysm size was 1.35 (95% CI, 1.05–1.72, *P* = 0.02). However, 4 other studies^[13,17,21,23]^ indicated that the aneurysm diameter was not significantly larger in the AAA patients with the MTHFR 677TT polymorphism than in those with the MTHFR 677CC polymorphism. Our previous study^[20]^ also showed no significant association between the MTHFR C677T allele and aneurysm diameter with no significantly higher increase in aneurysm size in patients with the CT or TT genotype than in those with the CC genotype. Interestingly, in our previous study,^[20]^ there was a significant association between the MTHFR 677T allele and the risk of AAA (OR = 2.46; 95% CI = 1.10–5.50; *P* = 0.03) in the subgroup of patients who consumed alcohol, indicating that consuming alcohol may play an important role in the association between the MTHFR C677T polymorphism and AAA. These results indicate that other factors may play a role in the association between the MTHFR C677T polymorphism and AAA. Additional studies focusing on the association between the MTHFR C677T polymorphism and AAA taking into account confounding factors and their interactions as well as subgroup analyses are needed to clarify these findings.

Our study provides evidence of the association between the MTHFR C677T polymorphism and AAA risk by performing a comprehensive meta-analysis. When interpreting our findings, several limitations should be considered. First, only 2 of the included studies analyzed Asian populations and no study data from ethnicities other than Caucasian and Asian populations were included. Thus, additional studies with large sample sizes designed to include other ethnicities are needed. Secondly, detailed information on individual patients in some studies was not available. Thus, we could not assess the risk of AAA according to stratification of other AAA risk factors. Gene–gene and gene–environment interactions were not fully resolved in this meta-analysis due to the lack of available data and could be addressed in future studies. Finally, our main analysis was based on unadjusted estimates of ORs owing to the lack of sufficient data. However, a more precise analysis should be conducted if adjusted estimates of ORs are available in all studies.

## 5 Conclusions

In summary, our present meta-analysis provides evidence of the association between the MTHFR C677T polymorphism and AAA risk, and suggests that the MTHFR T allele increases the risk of AAA in younger, nonsmoking, larger-AAA-diameter subgroups of patients. Future studies, including different subgroup analyses, are warranted to verify our findings.
